# Like clockwork? (Re)imagining rhythms and routines when living with irritable bowel syndrome (IBS)

**DOI:** 10.1111/1467-9566.13504

**Published:** 2022-08-05

**Authors:** Lauren White

**Affiliations:** ^1^ School of Education University of Sheffield Sheffield South Yorkshire UK

**Keywords:** body, crip time, IBS, irritable bowel syndrome, pace, routine, time

## Abstract

Temporal trajectories of health, illness and disability—from biographical change to micro‐embodied practices within social time—are important strands within medical sociology and disability studies. Drawing upon a UK‐based qualitative study using diaries and follow‐up interviews to explore everyday life with irritable bowel syndrome (IBS), this article explores routines when living with the condition. It focuses specifically on accounts of routines being anticipated, slowed down and stretched out to accommodate and/or care for bodies, with personal and social rhythms weaved in, out and with each other. Such reflections are told through participants’ accounts of knowing routines and rhythms, stretching out and pacing morning routines to care for the body and how everyday practices are reimagined as the body and the social meet. Drawing upon the concept of ‘Crip Time’ where the social bends to meet with the body, this article seeks to illuminate important intersections between medical sociology and disability studies through accounts of living with IBS. This article demonstrates the entanglement of structural, disabling temporal rhythms and embodied temporalities, through an acknowledgement of routines reimagined. It offers a contribution to both medical sociology and disability studies in reimagining social lives with embodied temporalities in mind.

## INTRODUCTION

Until I was about 26 or 27, I was as normal as anybody. One bowel movement a day, first thing in a morning, that was it. Then, it was two events. I’m not quite sure what caused it. I started running marathons, and that’s when I had my first problem. BUT at the same time, I was getting divorced. So, stress, running…that’s when it started, really. I’ve never experienced the pain that I know other people experience, but loose bowel movements, unpredictability, have always been an issue. *It’s a routine thing, really*.

(Terry’s interview, emphasis added)

In the quote above, Terry describes a life of ‘normal’, ‘regular’ bowel functioning, a ‘once a day, first thing in the morning’ scenario. What Terry then articulates is a change in circumstances, but alongside this change is an altered bowel, and a consequent shift in his daily routines. Terry’s account reflects what Bury (1982) identifies as biographical disruption—the life events and illness that shift an individual and their everyday structures. Yet for others, irritable bowel syndrome (IBS) and a ‘disrupted’ bowel have always been part of their biography. But how can we acknowledge change within the body together with an attentiveness to its embeddedness within social structures and time? Terry’s account brings us to the routines of the everyday. As this article will show, participants’ accounts include strategies or (re)negotiations of time involving the scheduling, flexing morning routines and negotiating social times with others. I argue that an appreciation of such routines is resonant of what we may see as crip time (Kafer, 2013)—a distinct offer of critical disability studies—which acknowledges the ways in which social clocks bend to meet bodies and minds.

This article builds upon a qualitative study exploring everyday life with the common health condition, IBS. Those with IBS can experience abdominal pain, bloating, constipation and/or diarrhoea and an urgency to use the toilet (NHS, 2022). Literature within the social sciences that has explored IBS has focused on the diagnostic and labelling processes (Dixon‐Woods & Critchley, 2000), what it means to live with an ‘unreliable’ body (Håkanson et al., 2009), navigating ‘unpredictability’ (Rønnevig et al., 2009) and processes of alterity and othering (Laursen et al., 2021). My research sought to shift attention away from accounts that focus solely on the health care or medicalised encounters, the body, and framings such as stigma and (un)containable bodies, focusing instead on *everyday,* structural encounters whereby IBS is experienced in *mundane* but *significant* ways.

Experiencing symptoms such as abdominal pain, constipation, diarrhoea or an urgency to visit the toilet requires a reimagining of time to balance bodily needs and structural accommodations or constraints. With such structural constraints and bodily needs in mind, this article focuses on the reimagining of routines through accounts of time being anticipated, slowed down or extended. This includes (1) attention to weekly routines and the anticipation of IBS; (2) mornings as a significant fragment in time and (3) embodied and social temporalities within the interdependent scheduling of everyday life. Together, such findings highlight the relevance of rhythms and (re)organisations to routine when living with IBS.

With these findings in mind, I look to bring together contributions from medical sociology and critical disability studies in thinking about changing times and recognising social temporalities with bodies in mind. This article looks to the concept of crip time (Kafer, 2013) as helpful in theorising temporalities of living with IBS. In appreciating and being attentive to the social relational model (Thomas, 2007) and arriving with critical disability studies (Goodley, 2014) through crip time, I wish to demonstrate how structural conditions meet with the body (and the bowel) in time. This article, and thus this theorisation, looks to assimilate contributions of embodied temporalities by thinking through crip time as worthy of attention to medical sociology and critical disability studies alike. Through the accounts of living with IBS, I hope to demonstrate the entanglement of structural, disabling rhythms and embodied temporalities, with a shared appreciation for a reimagining of social clocks for diverse bodies. These entanglements, I argue, require an intimate bringing together of medical sociology and critical disability studies, where the latter centres disabled bodies as a central concern.

## TEMPORALITIES IN MEDICAL SOCIOLOGY

Time is, in many ways, a key central conceptualisation in which experiences of health and illness have been understood. For instance, a longstanding temporal concept commonly returned to and drawn upon throughout medical sociology is Bury’s (1982) ‘biographical disruption’—a concept described to highlight changes to everyday life brought on by illness. However, there is the challenge to the idea of sudden disruption, accounting for health conditions and disability present throughout a person’s life or indeed always in fact part of their life and their biography (Monaghan & Gabe, 2015). Williams’ (1984; 2000) work on narrative reconstruction sought to offer an account of lives reshaped. Frank’s (1995) illness narratives is yet another central text in which illness and time come to be in relation to each other, with multiple narratives of chaos, restoration and quest thrown into temporalities. Such theorisations have sought to understand the ‘macro’ temporalities, biographical work and life changes, as opposed to the temporal *minutiae* of everyday routines and social lives. Scambler and Scambler (2010) argue that such concepts have stood the test of time within discussion of health, illness and disability. Indeed, such accounts lend themselves to Terry’s introduction, but an acknowledgement of lived rhythms and routines is another part of the story.

Beyond temporal theorisations within medical sociology described above, temporalities have been touched upon in understandings of individual bodies and their situatedness within social worlds. This is particularly the case with understandings of relationships between social environments (and their normative constraints) and how they come to meet with bodily necessities. Theorising the body within this has been particularly significant (Kelly, 1992; Kelly & Field, 1996), including literature on experiences on inflammatory bowel disease (IBD) (Kelly, 1992; Polidano et al., 2020; Saunders, 2014) which are, in some ways (when focusing on some similarities of societal needs and access as opposed to the diagnostic label), relevant to understandings of IBS. Theorisations within medical sociology include an appreciation of the social temporalities of bodies such as eating (Twine, 2015), sleep (Coveney, 2013; Meadows, 2005; Williams, 2011) and reproduction (Earle & Letherby, 2007; Jones, 2020). Within these contributions is a critique of normative understandings of bodily rhythms and what may be considered a ‘biological’ or body clock (Adam, 2004) through an acknowledgement of social, relational, material and temporal contexts in which they are situated. Temporalities within medicalised encounters have also been highlighted, such as accounts of waiting (Pedersen et al., 2021). Similarly, care is another example in which temporalities are illuminated, described as ‘process time’ where care takes ‘as long as it takes’ (Davies, 1994). As Buse et al. (2018) highlight, care is difficult to fix in time due to the unpredictability of bodies, care it needs and frictions within fixed structural institutional routines. An example often cited is ‘toiletry time’ (Inglis & Holmes, 2000) which is particularly relevant for understanding living with IBS.

More recently, Polidano et al. (2020) have explored how young people can experience ‘biographical renewal’ following stoma surgery and its liberating effects while living with IBD. However, such turns and awareness of the body are significant in considering the relationship between medical sociology and disability studies and the concept of crip time (Kafer, 2013; Sheppard, 2020) with the accounts presented later in this article. Similarly, Kathy Charmaz’s (1991) work ‘Good Days, Bad Days: The Self in Chronic Illness in Time’ is further influential and forces an engagement in temporalities of illness, capturing multiple and varied levels of temporalities at play—from biographical, life course narrative perspectives through to an understanding of the temporalities of everyday routines of care for the body. Whilst many have focused on more biographical dimensions to Charmaz’s work, the perhaps more mundane temporalities are particularly helpful to appreciating living and valuing illness and disability in time. For instance, Charmaz (1991) details a focus on everyday routines, slowing down, time stretched out and supported within broader relationships and interdependencies—flexibilities and recreations of time very much related to crip time (Kafer, 2013). Of course, many of the conceptualisations within medical sociology have been challenged within disability studies for their deficit or personal tragedy framing, with less focus on social oppression and active attempts in ensuring equality and access (see Thomas, 2021 for a detailed discussion of this). The social relational model developed by Carol Thomas has been a necessary gap here that accounts such tensions between social worlds and embodied personal lives (Thomas, 2007). Critical disability studies build upon this but see disability as a driving force and way of understanding and knowing with broader disruptive capacities (Goodley et al., 2019). Crip time further works as a generative perspective as part of this shift to crip the normative. This theorisation brings us to critical disability studies and crip time as a connecting and important theorisation and application in which living with a condition like IBS can be understood. Further to this, crip time facilitates an understanding and perspective to the broader social world.

## CRITICAL DISABILITY STUDIES AND CRIP TIME

As previously highlighted in articulations within medical sociology, threads of temporality have long been key areas of understanding and theorising disability. Within such theorisations have been an appreciation of time reworked and reconceptualised within broader socio‐material contexts and frameworks, and that acknowledge diverse bodies in relation to this. As defined in the introduction of this article, crip time is a clear example of appreciating such temporality. Kafer (2013) works at defining crip time as follows:

Crip time is flex time not just expanded but exploded; it requires reimagining our notions of what can and should happen on time, recognising how expectations of “how long things take” are based on very particular minds and bodies… Rather than bend disabled bodies and minds to meet the clock, crip time bends the clock to meet disabled bodies and minds.

(Kafer, 2013, p. 27)

In building up to such definition, Kafer (2013, p. 25) highlights how temporal categories are often used in constructions of disability and its response to temporal and medicalised terms such as ‘chronic’, ‘intermittent’, ‘frequency’, ‘relapse’ and ‘remission’. Samuels (2017) further unpacks crip time, suggesting the multiple ways in which it can be understood, including how linear, medical and normative trajectories are challenged, allowing for time to grieve for ‘lost’ time, acknowledging breaks in time where new rhythms of thinking and being in the world come to be, and the time afforded to be able to be sick. Temporalities have also been interwoven with disability studies whereby ‘non‐disabled’ and ‘able bodied’ have been recognised to be categories where ‘temporarily able‐bodied’ reminds non‐disabled people of the fleeting nature of such and that the abled/disabled distinction is, in fact, not fixed (Kafer, 2013). As Kafer (2013, p. 26) notes ‘familiar categories of illness and disability—congenital and acquired, diagnosis and prognosis, remission and relapse, temporarily able‐bodied and “illness, age, or accident”—are temporal; they are orientations in and to time, even though we rarely recognise or discuss them as such’. This temporality of everyday life is, for Snyder et al. (2002), ‘the fundamental aspect of human embodiment’. Of course, this is not to deny that for some, disability is more fixed and determined than for others and there are intersecting inequalities within this. Crip time is a flexibility and an expansion of time, both in response to bodily necessity and to societal barriers that make it so that more time may in fact be necessary.

Relevant to the experiences of IBS, Sheppard (2020) explores chronic pain and the entanglement of disbelief and uncertainty with pacing in understandings of crip time. Sheppard foregrounds understandings of crip time with the role of ‘non‐recognition’, as the first thing many of her participants shared, and as important in recognising the liminality and uncertainty when chronically in pain. For Sheppard (2020), she describes how for those with ‘undocumented disabilities’ (see Mollow, 2014, p. 185 who incorporates their own experiences of IBS as part of this description) whereby impairments are ‘neither visible nor definitively measurable by western medicine’, they experience ‘epistemic invalidation’ (Wendell, 1996) and thus, uncertainties regarding impairment or disability should be included in understanding crip time and its complexities within social worlds. This is something especially important to consider when appreciating the experiences of living with IBS—a common, but often contested and glossed over condition. That is not to medicalise such experience, but to highlight the role of recognition, diagnosis and labelling (Nettleton, 2006), in its relationship to everyday experiences of inaccessible environments and social attitudes.

Perhaps what is distinct to some medical sociology contributions is that crip time is an *intervention* in that it is a recognition that people arrive at, and require, different times (Price, 2011). Crip time, and crip studies more generally (Goodley, 2014), is a *challenge* to normative frameworks, through a destabilising of societal and cultural clocks. Samuels (2017) suggests that crip time is an act of resistance and a refusal to define or fit within regimented economic and cultural imperatives. Relevant to this article in her discussion of her bowel, Vidali (2010) promotes crip encounters by encouraging the reader to rethink containable and controllable bodies—the mundane moments of dinner time and disruption of conventions by asking to be excused to visit the bathroom. Such articulations are simultaneously seen as a refusal to participate and as a reminder for others to rethink normative expectations of embodiment. McRuer (2006) suggests that ‘one day all politics will be crip’, thus recognising the value of crip time within, and beyond, disability. This is necessary both in discussions within medical sociology and as a core concept in sociology more generally (Thomas, 2021). In explorations of living with IBS that will be detailed shortly, there are further opportunities for recognising how ‘toiletry time’ (Inglis & Holmes, 2000) can be reimagined with crip time, reflecting and responding to broader politics, economics and cultural contexts.

## THE STUDY

This article draws upon empirical findings from a qualitative research study exploring everyday life with the common health condition, IBS. As part of an attentiveness to the everyday, particular attention was paid to mundane temporalities and the rhythms and routines in which IBS is negotiated. To get at the everyday nature of IBS, the places people go, the routines of their day and the people and things that matter in everyday negotiations, this study utilised diary methods and follow‐up interviews (Zimmerman & Weider, 1977). Studies in health research and medical sociology have implemented diaries to examine individual experiences of illnesses over time (Elliot, 1997). Building upon the reflections of diaries as important for record keeping, temporality was threaded through the diary accounts. As Plummer (1983, pp. 17–18) notes, ‘each diary entry…is sedimented into a particular moment in time: they do not emerge “all at once”, but day to day strive to record an ever‐changing present’. Some diary data included dated and timed records, descriptions of times of day and the time that is taken for daily activities with IBS in mind.

Anyone who identified as living with IBS was invited to take part. This reflected a prioritisation of the social experiences of living and self‐identifying with IBS as opposed to a medical recognition, given the complexities of the IBS from processes of labelling and identification, health seeking and how it comes to be diagnosed through the medicalised exclusion of other conditions including (but not limited to) bowel cancer, coeliac disease and IBD. It also sought to appreciate the numerous ways in which the personal and diverse roots of the condition and accompanying experiences could be felt, recognised and understood through the participants themselves (see Mollow, 2014 on ‘criphystemology’—a term sought to validate lived experiences of the undocumented). The research also had an autobiographical grounding (Stanley, 1993) in that it was shaped through my own experiences of living with a bowel condition. The research study was advertised through health‐care charities, social media and personal networks. Twenty‐five people who identified as living with IBS took part in the project between 2017 and 2018. Ethical approval was granted from the University of Sheffield. Though scientific and medicalised knowledge highlight IBS as being twice as common in women than in men, I sought to challenge the gendered associations with IBS (Björkman et al., 2016) and with health seeking and diagnosis more generally (Annandale, 2014). I sought to challenge this by having purposeful recruitment for men who identified as living with IBS to allow for a gender‐balanced participant group. In the end, 16 women and 9 men took part in the study. Participants were predominantly White British, though an ethnically diverse sample would be encouraged in future research given well‐documented health inequalities. Given the participatory and open‐ended nature of the diary method and follow‐up interviews, specific demographic information such as age, social class, sexuality, ethnicity and disability were partially revealed on the terms of the participants preference for disclosure and self‐identification.

Participants were invited to complete their diaries via paper, electronic or audio, to allow for preferences and accessibility (White, 2021b). Guidance was provided, encouraging participants to respond to ‘Tell me about your day with IBS’ with specific prompts regarding their daily routines such as morning routines, factoring time into the day to account for going to the toilet, accommodating IBS more broadly and the temporalities of travel. Guidance suggested that participants keep a daily diary for approximately 2 weeks, although the entries were diverse in that some offered biographical narratives and others logged, in situ or reflective, daily navigations. Flexibility was offered when keeping diaries, both in terms of the regularity of diary keeping, how it could be written and the length of time the diary could be kept for. After completing the diaries, participants were invited to an interview with diary entries acting as prompts for discussion, as well as broader narrative questions about living with IBS and what mattered to them. Interviews lasted between 40 min and 3 h. Thirteen interviews were conducted in person (taking place in people’s homes, offices or local cafes) and 12 via telephone.

Following the open‐ended and diverse ways in which diaries were completed and participants’ experiences were articulated in the interview setting, both narrative (Riessman, 1993) and thematic analysis (Braun & Clarke, 2006) were utilised. Participants and biographical narratives derived from self‐written diaries directed a narrative analysis and the production of individual portraiture surrounding what was important to them and coming to live with IBS. A temporal attentiveness was also integral to the completion of diaries in the documentation of days, times and routines, evident in the accounts to follow. Similarly, questions surrounding time and routine were also asked as part of the follow‐up qualitative interview. Specific thematic analysis associated with the temporalities of living with IBS will be revealed in the pseudonymised accounts detailed in the upcoming accounts of (1) weekly routines and periodicity; (2) morning routines and (3) the temporal interdependencies of everyday social activities. Through the temporalities of living with IBS, the accounts draw specific attention to an appreciation and accommodation of time.

## RHYTHMS AND ROUTINES

Saturday: Like clockwork, after drinking wine the night before combined with an early start (a trigger), I get up and go straight to the toilet.

(Amy’s diary)

As emphasised in the title of this article and driven by Amy’s description, accounting and the expectation, symptoms of IBS came to her, ‘like clockwork’. Within many of the daily diaries and explanations was an awareness of IBS and how it situated itself, and indeed interacted with, normative working weeks. Despite what may be seen as ‘disruption’ to routine as a result of IBS, participants like Amy expressed the predictability as experienced within weekly routines and the embodied knowledge of when this happens, the reasons for it and how to respond. This embodied knowledge is a learning of the processes of eating, the temporalities of digestion, the time it takes for things to ‘work their way through’, toilet trips and being prepared spatially, temporally and materially for when may happen—in and out of ‘expected’ time. Lefebvre (2014, p. 29) highlights how the body serves us as a metronome by keeping to time and establishing rhythms. Of course, this is inclusive of the temporalities of impairment, or symptoms that are anticipated, cared for and responded to within the routines and rhythms of everyday life. As Samuels (2017) notes, crip time is broken time in that breaks in bodies and minds mean new rhythms, altered patterns of thinking, feeling and moving through the world to respond to bodily requirements. Of note were the rhythms of people’s individual weeks and the times at which IBS was most likely to affect them. For example, Shaun noted that Tuesdays were a significant day for him. His diary explained:

I can tell what I’ve done or what I’ve had on certain days. I know when it is going to come through. Tuesday is my worst day. That’s because I like to meet my friends on a Saturday evening, and I have more to drink than I should really. But it takes two days before I see any signs.

(Shaun’s interview)

Shaun describes what Kafer (2013) refers to as ‘anticipatory scheduling’, where the present moment comes to be measured against moments to come. Kafer (2013, p. 39) describes this in relation to conserving energy or anticipating pain and preserving the body against structural pressures. Crip time reads this as a practice of self‐care and accounting for *pleasure* and *agency* as opposed to productivity (Kafer, 2013). In many ways, Shaun’s knowledge of such scheduling and his commitment to the social despite future symptoms is in keeping with such ideas—a self‐care of the social and of participation.

Of course, within such change and responsiveness to the body and its requirement within rhythms and routines is how it is always in relation to, and situated within, structural conditions and expectations. This is particularly important for thinking about articulations of working lives and capitalist pressures of what this comes to look like. Returning to Amy, she explained how she had been comfortable in a job but keen to explore new opportunities. However, rhythms, routines and the comfort of access to toilets at work caused anxiety in pursuing such futures. She explained:

I have now been at my current job for a year and am getting to the stage where I am starting to think about looking for my next opportunity. However, one thing that stops me is the fact that I am so set up with my toilet routine at my current place of work. I know where the different toilets are, I know which ones are quiet, I know to take my phone with me, so it looks like I am going to make a call rather than dashing off for a lengthy toilet trip. It seems a ridiculous reason not to look for a new job, but I always remember my first job when I was 18. There was one small toilet for all the staff to use and it was not well ventilated. I worked 3 days a week—Monday to Wednesday. I would spend those days dosed up on Imodium and then the second half of the week taking laxatives as I was so constipated and then start again with Imodium on the Monday. It was a horrible way to live but I was newly diagnosed with IBS, in a new job and the toilets were not suitable to avoid embarrassment.

(Amy’s diary)

As well as the repeating rhythms of disruption, Amy’s working routine also brings to attention the social and environmental barriers associated with accessible public toilets (White, 2021a). Such narratives around work also sit within the pressures of employment and neoliberal ideals where bodies can never truly fit (McRuer, 2006) and thus must be compromised (Mitchell & Synder, 2015). This then results in individuals taking this on personally, being the bounded body within the workplace (Shildrick, 2015) and feeling prohibited from their social desires regarding their working lives. Relatedly, Carl explained how his IBS often came about when Monday morning arrives. He explained:

Say I work a standard five‐day week, I’ll be governed as to when and where I can go [to the toilet]. If I get two days off, I probably won’t go in those two days, because I know I can go when and where I want. When I know I’ve got to be somewhere at a certain time, then I’ll usually pay for it. I usually pay for it on a Monday morning…I know I’m going to be governed by time and travelling…I’ll get up 45 minutes before I catch the bus to work. I’ll go downstairs, have a coffee, smoke, and I’ll factor in getting all my gear together and going to the toilet before I have to go for the bus. There have been some mornings where I’ve been, I’ve got me coat on, and I’ve been just about to go out of the door, and I’ve thought, “no, I’m going to have to go again”, just as I’ve got out of the front door and the bus has gone.

(Carl’s interview)

Carl’s example of leaving the door for work is a particularly insightful example of temporal and structural pressures imposed, and indeed their impact on the body, but also how these can indeed shift when such pressures of the need to be ‘on time’ are removed. Mornings are often a ‘*time squeeze*’ (Southerton, 2003)—being ‘ready’ in a normatively limited time frame to be ready for work or activities of the day.

Accounts such as Caroline, Carl and Amy draw attention to how embodied knowledge and social expectations come from living with IBS, thinking about rhythms and routines within everyday contexts. For Amy, it is ‘like clockwork’, for Shaun, it is every Tuesday and for Carl, it is the Monday morning rush. Implicit within these accounts are how such weekly routines and changing bodies and bowels situate themselves within broader social structures. Building upon the example from Carl exploring Monday mornings, attention will now be turned to thinking about how IBS is accommodated within social lives and importantly, time flexed, specifically through examples of morning ‘routines’. By focusing on the morning routine and care for the body as part of this, the accounts highlight how the morning routine is changed—9 AM becomes 11 AM. More specifically, morning routines for individuals meet with, and respond to, structural expectations or responsibilities and vice versa. Time, and specifically morning routines, are thus (re)organised in living with IBS.

## THE ‘11 O’CLOCK RULE’

As seen within Amy’s account of her working week and Carl’s rush to work, their accounts drew attention to ‘anticipatory scheduling’ (Kafer, 2013, p. 39) and the important intersection with inaccessible social environments and temporal pressures. Further to these temporalities must be a recognition of how these are responded to—how long things take, slowing things down and stretching time when necessary. Crip time is a recognition of how long things take, inclusive of flexible and expansive time, both in response to bodily necessity and in response to societal barriers that make it so more time is in fact necessary (Kafer, 2013). In discussing her everyday experiences of living with IBS, Caroline described the importance of taking things slowly in the morning:

I suppose I’ve never had a kind of routine where you have breakfast and go to the loo before going to work. I do things quite slowly in the morning, so I have to get up quite early because I often go quite dizzy, So I allow myself extra time to wake up quite naturally before I jump out of bed.

(Caroline’s interview)

Caroline explained that she has never had a routine of breakfast and going to the toilet before heading off to work. However, the importance of pacing and slowing down is highlighted here. Charmaz (1991, p. 161) talks about the intricate trade‐offs of ‘juggling and pacing’, recognising that often maintaining a job call this into question. Caroline recounted missing work a lot, taking days ‘off sick’ to ‘recharge her batteries’ or working from home to cut out the stress of commuting to work without a toilet. Caroline revealed feeling bad that she has ‘the highest amount of sick time off’ and how she felt frustrated at this. As Sheppard (2020, p. 43) notes, ‘Pacing can be an ableist rejection of chronic pain and fatigue, but also a crip embracing of living with chronic pain and fatigue. Pacing can be a site of conflict, of internalised ableism simultaneously. As a way of moving in/through time, pacing is both normative and non‐normative, read in opposing ways at once’. This is thus relevant to Caroline’s account.

In accounting for these pressures of *‘squeezed’* time (Southerton, 2003), getting up *earlier* became a prominent feature of extending time in the morning, before leaving the home to go to work or to social events planned for the day ahead. For many of us, rising in the morning and having breakfast is often when bowel movements occur. This makes mornings significant and important. Molly explained how she allocated extra time for her morning routine:

If I know that we’re going out early, I will get up a couple of hours earlier, purposely so I’ve been to the toilet before I go anywhere. Say I’m going to pick my friend up at quarter past nine to go swimming, I’ll get up earlier, so I know that I’ve been. You feel safer because you’ve already been. Sometimes you think you’ve been and then you go again before you go out. But I think if you’ve been before you go out, then you do feel that bit safer.

(Molly’s interview)

Molly and I talked about having ‘*already been*’ and what that means for the rest of the day. There is a security of *‘going’* (to the toilet) in the morning *and* in your own home, working as an assurance measure for starting the day. As Twigg (2006, p. 119) explains, ‘Washing, dressing, drinking, eating, excreting mark out and punctuate the day, giving it rhythm and structure; much of our sense of ontological security derives from this bedrock of bodily comfort, comportment and care. It creates routine and regularity at a directly physical level’. Certainty is gained from regular routines and time for toiletry practices and are made evermore important for a diversity of bodies within social worlds.

In highlighting the preference for these routines in the morning and at home, participants also acknowledged how they *extended* morning routines to account for their IBS. A striking example was from Joyce, who coined her morning with the ‘11 o’clock rule’:

Had an interesting thought today as I used to have the “eleven o’clock rule”. My family used to know that no matter how bad I was in the morning, especially early morning, I would be much better and able to face the day by eleven.

(Joyce’s diary)

Joyce and I discussed what the 11 o’clock rule meant for her:

The thing I found difficult and still find difficult is that I can get the same violent pains, which I wake up with every morning. I wake up every morning with pain and then I don’t know whether it’s going to just wear off or it’s going to land me in trouble. That’s where your first uncertainty comes. I think it’s that uncertainty that is hard. My family has the 11 o’clock rule. If I was really bad in the morning, my daughter would ring me up and say, “Well mum are you coming to us or not? or are you coming on the later train?” and I could pretty well guess as to which way it would be.

(Joyce’s interview)

The 11 o’clock rule is crip time—a bending of the social to meet with the body. The 11 o’clock rule is expanded time and a collective reimagination of how long things take with Joyce’s body in mind (Kafer, 2013). Joyce’s personal time in line with her morning IBS symptoms now meets with the recognition from her family. Charmaz (1991, p. 172) highlights that ‘temporal incongruence results when intimates do not share similar ways of thinking about and structuring time’. However, Joyce’s family meet with this restructuring of her morning, finding an alternative practice for her to participate in the day. Julie also identified 11 o’clock as a time for her IBS to have settled down. Prior to retiring, Julie had to wake at 5:30 AM to get ready for work, building in *‘extra time’* to account for the flare of IBS. Since retiring, she now schedules any appointments for 11 o’clock onwards. Julie also describes how the removal of the ticking clock of the time pressures from work has changed her IBS. Again, this reminds us of the importance of how a societal clock intersects with the body. Building upon Joyce’s account of family working to change the clock, attention will now be turned to social and interdependent time, as reimaginations of routine and time intertwine with others.

## SOCIAL TIMES AND TEMPORAL INTERDEPENDENCIES

In the previous section, accounts such as Joyce’s demonstrated how time comes to be flexed (Kafer, 2013) to meet with the body, made visible through her description of the ‘11 o’clock’ rule created with her family. Together with the stretching of time, this example highlighted how negotiating time with a condition such as IBS is also situated within relationships and care. For example, and perhaps related to previous accounts around mornings as a crucial segment of time, Terry described how he sticks to a strict routine. Further to this, he explained how such rhythms and routines are situated as social time (Adam, 2004) and within the context of interpersonal relationships:

My morning routine is the same. Part of managing the condition is sticking to a strict routine. This has become easier since I retired. When I was at work, it was far more difficult to manage. I often had to chair lengthy meetings and was often anxious about the possibility of having to leave the meeting to go to the toilet. For many years now, I have never scheduled any appointments or activity around teatime because I usually require a bowel movement at that time. My partner obviously understands this and fits in with this routine.

(Terry’s diary)

Much of Terry’s account, such as the teatime activity, relates to points highlighted earlier where an emphasis on anticipatory scheduling is necessary to account for symptoms (Kafer, 2013, p. 39) as situated within and in response to the social, but there is an important dimension of the *intertwining* of time with others to draw attention to. In her book, ‘In the Meantime’, Sharma (2014) explores the concept of ‘temporal interdependence’.^1^ While her focus is on the interdependencies and power relations between workers and whom they may give their time and attend to, interdependence is a concept relevant within critical disability studies, as *central* in what it means to be human (Goodley, 2020, p. 57) and is thus helpful in thinking about living with, and negotiating, temporalities of IBS. Thus, in Terry’s example, ‘anticipatory scheduling’ (Kafer, 2013, p. 39) intertwines with ‘temporal interdependence’ (Sharma, 2014)—routines are anticipated and navigated, *together*. In a different vein, Ellie discusses the temporal intersections between herself and colleagues at work. She described her previous work where such temporal scheduling and ‘cover’ is significant in allowing time for going to the toilet:

I used to work on a TV show, and I was a floor manager and couldn’t leave. But obviously some days I would just have to so I would say over the Headset “Could someone please come into the studio?” Then I would have to say to someone please “Can you cover me for 5 minutes while I go to the toilet?” …If I know I’m going to have a bad day I may tell my boss at the beginning of the day, hey I may need you to come and cover for me because if I run in at some point and they will say it’s fine.

(Ellie’s interview)

Again, Ellie’s account reiterates the importance of having control over one’s work environment, the structuring of working time and the time allowed to leave for a toilet break (Inglis & Holmes, 2000). As with flexibility in the morning routine and the pace of such, being able to take time out is made visible.

In keeping with the fusing of scheduling (Kafer, 2013, p. 39) and temporal interdependencies (Sharma, 2014), an everyday routine that frequently came to be discussed was dog walking. In caring for the toiletry needs of one’s pet, there is often a recognition that one’s own toilet access may not be accessible in this social context. Walking the dog often requires visiting a green space where accessible toilets are often not present. As mentioned earlier, Terry’s daily routine had changed since retiring, setting his own routine and not that of working expectations. Walking his dog Bertie is part and parcel of his everyday routine, and Terry’s and Bertie’s routines now work together. He explained:

I factor in what time I get up and what time I need to go to the toilet before I take him [Bertie] out. I make sure I go twice before I take him out in the morning. I wouldn’t dream of not doing that. I wouldn’t be able to. I always take him out and get back by about half‐past four just in case.

Lauren: I suppose it’s a routine, isn’t it?

Terry: It’s just there. It’s automatic; it’s just what I do and part of my life. It’s become that over the years because it’s been going on for so long, really.

(Terry’s interview)

Building upon the introductions of Terry, his walking routine with his dog Bertie is now an automatic practice of everyday life. This demonstrates how the social world and environment come to intersect and meet with the bodies (or bodies when being inclusive of non‐human companions). Thus, routines with others, both anticipated and actualised, intertwine and accommodate each other in the embodiment of everyday social lives.

## DISCUSSION AND CONCLUSION

With our lives running on abstract time, from the hours of sleep and waking, to mealtimes, work and private life, we force our bodies to fit a rhythm that is not our own.

(Bates, 2019, p. 81)

As noted by Bates in the quote above who builds upon people’s accounts of living with illness, individual bodies are forced to fit within structural rhythms that are not their own. And yet, bodies and structures bend back and forth in the accounts of living with IBS through accounts of rhythms and routines. Perhaps most importantly here, and in thinking with crip time (Kafer, 2013), bodies resist rhythms and recreate their own, together with others, in their weekly routines, relationships and everyday lives.

As introduced at the start of this article, temporalities have been a longstanding thread within medical sociology and disability studies. This includes biographical, life course understandings through to temporal (re)arrangements of everyday practices associated with the body in social life. While contributions within medical sociology have often been seen to frame experiences that deploy negative connotations with illness or disability, highlighting social practices with bodies and social routines, and their entanglements, is significant. Within disability studies, attention on social structures and oppression have recognised how the social must meet with diverse bodies. In bringing in crip time (Kafer, 2013) that forces the body and the social in direct entanglement with each other, dialogues between disability studies and medical sociology that illuminate temporality are brought to attention (Thomas, 2021). Distinct to crip time is the nature in which change is prompted and provoked within people’s everyday lives and necessities for their bodies. Through the accounts of living with IBS, routines are anticipated, reimagined, stretched out and met with the social lives of others.

First, those living with IBS described the embodied knowledge and anticipatory scheduling (Kafer, 2013) within their weekly rhythms and routines. The weekly routines and awareness of such captured the anticipated symptoms and the regularity of such. Importantly, this was described as ‘like clockwork’ or anticipated on particular days that coincided with social structures such as social time and working pressures (Mitchell & Synder, 2015). This also offers a broader contribution by stressing the importance of how the taken‐for‐granted and personal, embodied routines, such as Amy and Carl’s, fits with the public and structural working schedule. Namely, the pressures of getting to work ‘on time’, how time is *squeezed* (Southerton, 2003) and inextricably bound up with the rhythms of our bodies (and bowels), and structures should recognise this.

Second, and building upon embodied knowledge and the scheduling of everyday practices, this article explored what participant Joyce called the ‘11 o’clock rule’. 11 o’clock is a manifestation of time flexed and the social meeting with the needs of personal bodily requirements (Kafer, 2013) or what some may describe as pacing (Charmaz, 1991; Sheppard, 2020). Molly described extending the morning routine by getting up earlier, demonstrating time and routine needed for caring for bodies, in their own space, in their own time (Twigg, 2006) and as significant and thus changing the temporal rhythms of everyday life. Importantly within this section is an attention to the stretching and flexing of routines that are met with social lives, care and relationships.

Third and finally, the accounts from those living with IBS highlighted how everyday practices were negotiated and met with social times, relationalities and other human and non‐human bodies. From meeting friends for lunch to colleagues stepping in to give us time, this section revealed the interdependencies of bodies and social lives when time is organised, and most importantly, flexed and stretched when necessary (Kafer, 2013). A particularly prominent example of this, where the ‘toiletry time’ (Inglis & Holmes, 2000) and negotiation between bodies came to the fore, was participants’ descriptions of walking their dogs. Timing dog walking in line with bodily necessities revealed not only the ‘anticipatory scheduling’ (Kafer, 2013) but the ‘temporal independencies’ (Sharma, 2014) between negotiated social times with others. For those with IBS, the mundane activities such as the early morning rises and walking the dog are anticipated (re)structured and (re)imagined in line with inaccessible public landscapes and (un)predictable bowels.

Finally, beyond intersections between medical sociology and disability studies is the application of crip time for sociologists more broadly. In thinking through intersections between medical sociology, disability studies and the role of crip time, there is a broader point to be made about ‘toiletry time’ (Inglis & Holmes, 2000) and bodily needs and necessary flexibilities in social time (Adam, 2004). As Goodley (2016, p. 157) notes that one ‘starts with disability but never ends with it’ as it becomes ‘*the* space from which to think through politics for all’. Cripping toiletry time has a broader relevance—from the gig economy worker unable to find time to go to the toilet to those provided with necessary time for care needed to attend to their bodies. A recognition of whose clock we are on, and how we might *change* it, is important for us all.

## AUTHOR CONTRIBUTION

The lead author is the sole contributor.

## Data Availability

Research data are not shared.

## ENDNOTE

^1^
In exploring ‘temporal interdependence’, Sharma (2014) describes taxi drivers embodied work in serving passengers and the strategies they exercise to ensure they keep to time. This includes restricting fluids to avoid taking time to use the toilet.
